# Some Non-Obvious Consequences of Non-Extensiveness of Entropy

**DOI:** 10.3390/e25030474

**Published:** 2023-03-09

**Authors:** Grzegorz Wilk, Zbigniew Włodarczyk

**Affiliations:** 1 National Centre for Nuclear Research, Department of Fundamental Research, 02-093 Warsaw, Poland; 2 Institute of Physics, Jan Kochanowski University, 25-406 Kielce, Poland

**Keywords:** non-additive entropy, Tsallis distribution, multiparticle production processes

## Abstract

Non-additive (or non-extensive) entropies have long been intensively studied and used in various fields of scientific research. This was due to the desire to describe the commonly observed quasi-power rather than the exponential nature of various distributions of the variables of interest when considered in the full available space of their variability. In this work we will concentrate on the example of high energy multiparticle production processes and will limit ourselves to only one form of non-extensive entropy, namely the Tsallis entropy. We will discuss some points not yet fully clarified and present some non-obvious consequences of non-extensiveness of entropy when applied to production processes.

## 1. Introduction

Entropy plays an important role in the study of the production mechanism of elementary particles observed in hadronic and nuclear collisions. This is the case both in the modelling of these processes based on thermodynamics (that is, on the description of distributions of all kinds of observables characterizing multiparticle production processes) and in their description in the language of statistical models (i.e., mainly on the description of their multiplicity distributions).

Over time, more and more new experimental results appeared, which began clearly to indicate that the originally used Boltzmann entropy (in the first case) or Shannon entropy (treated as a measure of information in the second), did not describe the results in the entire range of measured values. Experimentally observed distributions depart from the expected exponential form (in the first case) and from the Poissonian distribution (in the second) [1,2]. This was generally taken as an indication that different mechanisms operate, resulting in the occurrence of various types of correlations and fluctuations, and these do not fit into the scheme of equilibrium thermodynamics or the Shannon information measure [3]. This meant that it was necessary either to add appropriate conditions to the definition of the Boltzmann-Shannon entropy used, or to extend the very concept of entropy so that in its new form it could be applied to more complex systems without any additional conditions (their operation would be replaced by a new form of the entropy formula and by some new parameters appearing in it).

A multitude of new definitions of entropy and related measures of information have appeared in various fields of science (see, for example, [3,4,5,6,7] and references cited therein). In most cases, their distinguishing feature is their non-extensiveness. Here we will consider only the case of Tsallis entropy [5] $S_{q}$, which for q=1 becomes Boltzmann-Shannon entropy, $S=S_{q=1}$:

(1) $$\begin{array}{ccc} S_{q} & = & -\int dxf\left(x\right)\ln_{q}f\left(x\right)=-\frac{1}{1-q}\int dxf\left(x\right)\left[1-f^{q-1}\left(x\right)\right]\\ & & \;\overset{q\to 1}{⟹}\;S=-\int dxf\left(x\right)\ln f\left(x\right), \end{array}$$

which is currently the most widely-used to describe the particle production processes mentioned above (in fact, Tsallis entropy was introduced independently before and then rediscovered by Tsallis in thermodynamics [8,9]. It should be mentioned that from the point of view of information theory, the entropies $S=S_{q=1}$ and $S_{q}$ are related to a different, specific way of collecting information about the object of interest [10]. This observation has recently been used in cognitive science [11]). The reason for this is the quasi-power nature of the Tsallis distribution $f_{q}\left(x\right)$ that is obtained from it,

(2) $$f_{q}\left(x\right)=\exp_{q}\left(-x\right)=\left(2-q\right){\left[1-(1-q)x\right]}^{\frac{1}{1-q}}\;\overset{q\to 1}{⟹}\;f\left(x\right)=\exp\left(-x\right),$$

and, as it was shown a long time ago in [12,13,14], it is this type of distribution that is most suitable for describing the distributions of various variables in the full observable range of their variability. In fact, there are a variety of systems that do not comply with the standard equilibrium theory and that fit under the description of non-extensive entropy, thus suggesting that the entropic index *q* could be a convenient manner for quantifying some relevant aspects of complexity [5].

The Tsallis distribution is obtained by maximizing the Tsallis entropy using some constraints imposed on the distribution function sought. It turns out that in the commonly used version this procedure leads to a rather surprising result, namely that the non-extensiveness parameter *q* appearing in the definition of entropy is, in a sense, dual to the non-extensiveness parameter $q^{\prime }$ obtained from the description of the observed distributions. As we show in (Section 2), this result is confirmed by the simultaneous analysis of multiparticle production processes in nucleon and nuclear collisions. In (Section 3) we show how by properly redefining the functions $\exp_{q}\left(x\right)$ and $\ln_{q}\left(y\right)$ this problem of duality can be avoided.

Tsallis entropy $S_{q}$ is nonadditive, namely

(3) $$S_{q}\left(AB\right)=S_{q}\left(A\right)+S_{q}\left(B\right)+\left(1-q\right)S_{q}\left(A\right)S_{q}\left(B\right),$$

where *A* and *B* are two systems independent in the sense that f(AB)=f(A)f(B) and the parameter *q* is simply a measure of the degree of this non-additivity (note that we tacitly assume here and in all subsequent considerations that *q* is the same in both systems). If, hypothetically, we extended this reasoning to the system of ν independent components (again, with the same *q*), $A_{1}$, $A_{2}$, ..., $A_{\nu }$ such that $f\left(\prod_{i=1}^{\nu }A_{i}\right)=\prod_{i=1}^{\nu }f\left(A_{i}\right)$, then we would have some kind of non-linear non-additivity (in parameter *q*), because now

(4) Sq∏i=1νAi=∑i=1ννi(1−q)(i−1)∏j=1iSqAj.

To better understand the role of the parameter *q*, let us additionally consider the non-additive versions of conditional probability and conditional entropy. Let us say that the considered system can be divided into two subsystems, *A* and *B*, and that $p_{ij}\left(A,B\right)$ is the joint normalized probability of finding *A* in state *i* and *B* in state *j*. Then the conditional probability *B* with *A* being in the i−th state, $p_{ij}\left(B|A\right)$, is given by Bayes’ multiplication law,

(5) $$p_{ij}\left(A,B\right)=p_{i}\left(A\right)p_{ij}\left(B|A\right),$$

and the corresponding conditional Shannon entropy is

(6) S(A,B)=S(A)+S(B|A).

By analogy to Equation (3) we can now write the corresponding conditional non-additive Tsallis entropy as

(7) $$S_{q}\left(A,B\right)=S_{q}\left(A\right)+S_{q}\left(B|A\right)$$

where

(8) $$S_{q}\left(B|A\right)=S_{q}\left(B\right)\left[1+\left(1-q\right)S_{q}\left(A\right)\right]$$

(note that because $S_{q}\left(B|A\right)\leq S_{q}\left(B\right)$ one must have q≥1). This allows us to interpret the nonextensivity parameter *q* in terms of the conditional entropy as

(9) $$q=1+\frac{S_{q}\left(B\right)-S_{q}\left(B|A\right)}{S_{q}\left(B\right)S_{q}\left(A\right)},$$

and turns out to be crucial for nonadditive (quantum) information theory [15].

In practical applications, the non-extensiveness of the entropy manifests itself in the quasi-power character of the distributions obtained from it, i.e., in the case considered here in the appearance of the non-extensiveness parameter *q* in the Tsallis distribution. However, there is a problem here that we discuss in Section 2 and Section 3, namely that for a certain type of constraints, the parameters *q* in the definition of entropy and $q^{\prime }$ in the Tsallis distribution are not identical but dual to each other, i.e., $q+q^{\prime }=2$. Usually, the meaning of the non-extensiveness parameter is related to Tsallis distributions rather than to entropy as above. These, in turn, can be obtained in many ways, depending on the details of the described physical process and even from the Shannon entropy, if only the appropriate constraints are applied. We discuss this issue in more detail in Section 4. Section 5 contains our summary and conclusions.

## 2. From Tsalis Entropy to Tsalis Distribution

The Tsallis distribution (2) (valid for 0≤x<∞;1≤q≤3/2) is obtained by maximizing the Tsallis entropy (1) using the following constraints [16]:

(10) $$\int dxf\left(x\right)=1;\;\int dxxf^{q}\left(x\right)={\langle x\rangle }_{q}.$$

**Figure 1:**
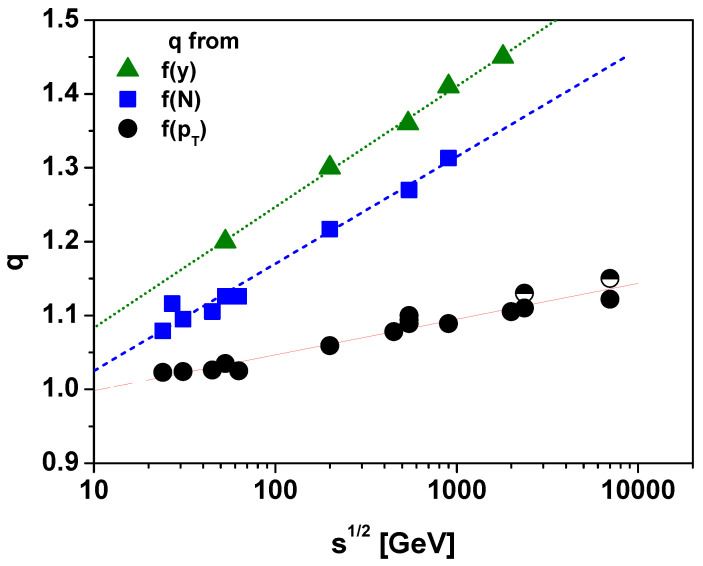
(Color online) Energy $\sqrt{s}$ dependencies of the parameters *q* obtained from different observables. Squares: *q* obtained from multiplicity distributions f(N) [17,18] (fitted by $q=0.88+0.063\ln[\sqrt{(}s)]$). Circles: *q* obtained from different analyses of the transverse momenta distribution $f(p_{T})$. Data points are, respectively, from a compilation of p+p data (full symbols) [19], from CMS data (half filled circles at high energies) [20,21] (fitted by $q=0.95+0.021\ln[\sqrt{(}s)]$. Triangles: *q* obtained from analyses of rapidity distributions f(y) [22,23] (and fitted by $q=0.92+0.071\ln[\sqrt{(}s)]$.

In most cases, it is this form of distribution that is used phenomenologically to describe the various distributions measured in high-energy multiple particle production experiments (with x=X/T and the scaling factor *T* is usually identified with the temperature and *X* denotes the energy or momentum of the measured particles; it also appears in the normalization as 1/T). As shown in Figure 1, using this form of Tsallis distribution one obtains from measurements of different observables (rapidity, multiplicity and transverse momentum) and for high enough energies $q^{\prime }>1$ (for low energies, conservation laws are important and they can sometimes push the parameter $q^{\prime }$ to the $q^{\prime }<1$ region). In addition, note that the values of *q* obtained from different observables are different (but always $q^{\prime }>1$). These differences are due to the influence of two factors. The first is whether $q^{\prime }$ is estimated from the temperature fluctuations obtained from data already averaged over other fluctuations or from data taking other fluctuations into account as well, and the second is that in different analyzes $q^{\prime }$ is obtained in other regions of the phase space.

However, this is not the only possible choice of constraints. Instead, using constraints in the form which seems to be more natural from the point of view of physical interpretation, namely that

(11) ∫dxf(x)=1;∫dxxf(x)=〈x〉

obtain [16]

(12) $$f\left(x\right)=q^{\prime }{\left[1-(1-q^{\prime })x\right]}^{\frac{1}{q^{\prime }-1}};\;0\leq x<1/\left(1-q^{\prime }\right);\;1/2<q^{\prime }\leq 1.$$

These two different definitions pertain to two different schemes of the nonextensive statistical mechanics [24]. It should be noted that [25] proposes a parametric technique that shows the equivalence of different schemes (including those discussed here), and [26] once again shows the relationship of both averaging schemes (i.e., Equations (10) and (11)) with duality q↔1/q. Now note that for

(13) $$q^{\prime }=2-q,$$

distribution f(x) from Equation (12) becomes f(x) from Equation (2) (note that in addition to the additive duality represented by Equation (13), multiplicative duality, q↔1/q, was also considered [27,28] shows the potential physical application of a combination of both types of duality to study cosmic ray physics). This means that the imposition of these constraints leads to a situation in which the non-extensiveness parameter *q* appearing in the definition of entropy is dual to the non-extensiveness parameter $q^{\prime }$ obtained from describing the observed distributions. The problem of this duality has been raised many times (for example in [29,30,31]), but it does not seem to have been put to the experimental test yet, at least not in the field of multiparticle production. It turns out, however, that experiments measuring the multiplicities and distributions of particles produced in nuclear (AA) and nucleon (nn) collisions are very useful for this purpose, because they simultaneously measure the multiplicities (enabling the estimation of the entropy produced) and particle distributions, and thus allow for the simultaneous determination and comparison of the non-extensiveness of the above mentioned relevant parameters and to verify the hypothesis of their duality.

Nuclear collisions are usually described by increasingly complex statistical models that try to account for all possible collective effects [32,33,34]. Because, however, for our purposes, the mutual relation between the entropies of AA and nn collisions will be important, to estimate the entropy in the nuclear collision, it will therefore be more convenient to use the phenomenological description based on the assumption that it can be described by a certain superposition of collisions of single nucleons (taking into account only nucleons that collided at least once and assuming that their collisions are independent—these are the so-called “wounded nucleons”) [35]. (The reason for this choice may be the fact that, despite its apparent simplicity, this model is still able to describe a surprisingly large number of experimental results [36,37]).

In this approach, the total observed multiplicity *N* is the sum of the multiplicities $n_{i=1,...,\nu }$ of particles emitted from ν individual sources, and the average total multiplicity 〈N〉 is the product of the average number of sources, 〈ν〉, and the average multiplicity from the source, $\langle n_{i}\rangle$, (which here is assumed to be the same for each source):

(14) $$N=\sum_{i=1}^{\nu }n_{i},\;\mathrm{and}\;\langle N\rangle =\langle \nu \rangle \langle n_{i}\rangle .$$

The identity of the sources assumed here means that their entropies are equal, so using the relationship (4) the entropy ν of such sources is

(15) Sq(ν)=∑k=1ννk(1−q)(k−1)Sq(1)k=1+(1−q)Sq(1)ν−11−q.

In further considerations, ν will denote the number $N_{P}$ of nucleons of the incident nucleus participating in the collision (i.e., participants), and $\nu =N_{W}/2$, where $N_{W}$ is the number of wounded nucleons. Continuing in the same vein and assuming that the total entropy is proportional to the average multiplicity of particles produced in the collision,

(16) S=α〈N〉,

we can relate the average multiplicities in nuclear (AA) and nucleon (NN) collisions, namely

(17) $$\alpha {\langle N\rangle }_{AA}=\frac{{\left[1+\left(1-q\right)\alpha {\langle N\rangle }_{pp}\right]}^{N_{P}}-1}{1-q}.$$

This simple dependence already allows for some preliminary assessment of the *q* parameter. It turns out that the observed $N_{AA}$ grows non-linearly with $N_{P}$, ${\langle N\rangle }_{AA}>N_{P}{\langle N\rangle }_{pp}$ [38]. Considering this observation from the point of view of entropy, it is clear that we must have q<1 here.

However, this is only a very rough estimate, because, strictly speaking, formula (17) is not fully correct with respect to the $S_{q}$ entropy. We will therefore return to Equation (15) denoting now the entropy for the whole particle production process by *s* and the corresponding non-extensiveness parameter by $\tilde{q}$, and their equivalents for nucleon collisions by *S* and *q*, respectively. The relation (15) for *N* particles now looks like this:

(18) $$s_{\tilde{q}}^{\left(N\right)}=\frac{{\left[1+\left(1-\tilde{q}\right)s_{\tilde{q}}^{\left(1\right)}\right]}^{N}-1}{1-\tilde{q}}\overset{\tilde{q}\to 1}{⟶}N\cdot s_{\tilde{q}}^{\left(1\right)}=\alpha N$$

where $s_{\tilde{q}}^{\left(1\right)}=\alpha$ is the entropy for a single particle. In the A+A collision with ν nucleons participating Equation (15) results in

(19) $$S_{q}^{\left(\nu \right)}=\frac{{\left[1+\left(1-q\right)S_{q}^{\left(1\right)}\right]}^{\nu }-1}{1-q}$$

where $S_{q}^{\left(1\right)}$ is the entropy for a single nucleon. Denoting multiplicity in single N+N collisions by *n*, one can write that the respective entropy is

(20) $$S_{q}^{\left(1\right)}=S_{\tilde{q}}^{\left(1\right)}=\frac{{\left[1+\left(1-\tilde{q}\right)s_{\tilde{q}}^{\left(1\right)}\right]}^{n}-1}{1-\tilde{q}},$$

whereas the entropy in A+A collisions for *N* produced particles is

(21) $$S_{\tilde{q}}^{\left(N\right)}=\frac{{\left[1+\left(1-\tilde{q}\right)s_{\tilde{q}}^{\left(1\right)}\right]}^{N}-1}{1-\tilde{q}}.$$

This means therefore that

(22) $$S_{\tilde{q}}^{\left(N\right)}=S_{q}^{\left(\nu \right)}.$$

Parameters *q* and $\tilde{q}$ are usually not the same. However, from analyzes in [38,39] one obtains that for NN collisions (where $N_{P}=1$) $\tilde{q}=1$. On the other hand, for $\tilde{q}=q$ Equation (22) corresponds to the situation encountered in superpositions as now one obtains

(23) $${\left[1+\left(1-q\right)s_{q}^{\left(1\right)}\right]}^{N}={\left[1+\left(1-q\right)s_{q}^{\left(1\right)}\right]}^{n\nu }\;\mathrm{or}\;N=n\nu .$$

**Figure 2:**
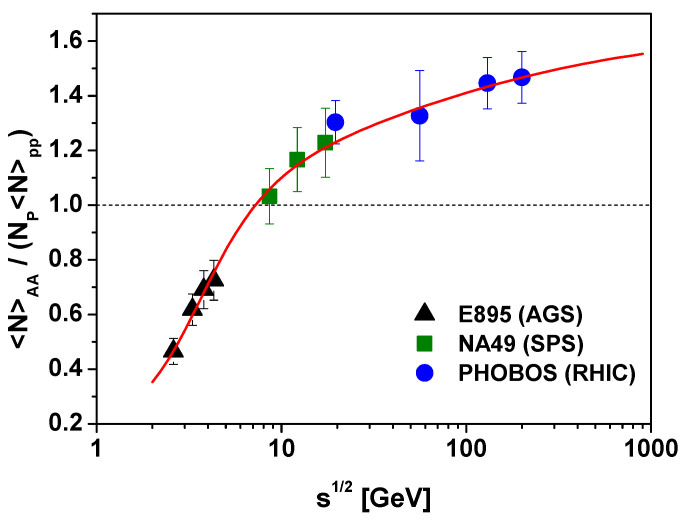
(Color online) Energy dependence of the charged multiplicity for nucleus-nucleus collisions divided by the superposition of multiplicities from proton-proton collisions using Equation (24) with $c_{2}=1.7$ and with $c_{1}$ depending on energy $\sqrt{s}$ according to $c_{1}\left(s\right)=1.0006-0.036s^{-1.035}$. Experimental data on multiplicity are taken from the compilation of Ref. [41].

In the general case, we obtain the formula for the ratio N/(ν·n))

(24) $$\frac{N}{\nu \cdot n}=\frac{1}{\nu n\cdot \ln c_{1}}\ln\left[\frac{{\left(c_{2}c_{1}^{n}+1-c_{2}\right)}^{\nu }-\left(1-c_{2}\right)}{c_{2}}\right],$$

where

(25) $$c_{1}=1+\left(1-\tilde{q}\right)s_{\tilde{q}}^{\left(1\right)};\;\;c_{2}=\frac{1-q}{1-\tilde{q}},$$

which for $N=\langle N_{AA}\rangle >$, $n=\langle N_{pp}\rangle$ and $\nu =N_{P}$ is presented in Figure 2 for different reactions (see [40] for more details). Note that for energies $\sqrt{s}>7$ GeV one has $c_{1}>1$. This means that $\tilde{q}<1$ and (because $c_{2}>0$) also q<1, confirming therefore previous estimates based on Equation (17).

This, however, is as much as can be said for sure, because while the distributions can give exact values of the parameter $q^{\prime }$, the same cannot be said about *q* except that q<1 (at least in a certain energy range). We still have too many free parameters here, e.g., unknown a priori entropy $s_{q}^{\left(1\right)}$. Therefore, while the statement that mostly we have $q^{\prime }>1$ and q<1 seems certain, it is not known how exactly (if at all) the duality $q^{\prime }+q=2$ (13) is satisfied.

## 3. More Thorough Screening of Duality

**Figure 3:**
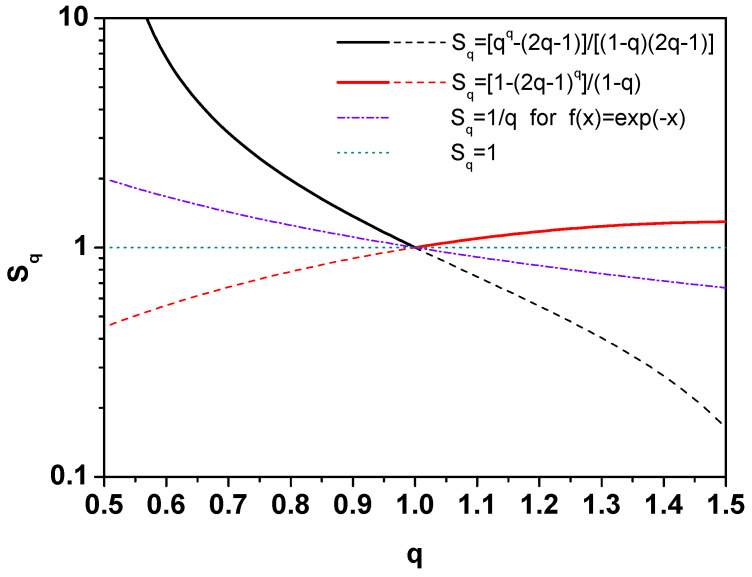
(Color online) Tsallis entropy for different nonextensivity parameter (see text for details).

We will now deal with the problem of duality in more detail. Figure 3 shows the entropies $S_{q}$ obtained from the distributions (12) for $0.5<q^{\prime }\leq 1$,

(26) $$S_{q}=\frac{q^{q}-\left(2q-1\right)}{\left(1-q)(2q-1\right)},$$

(here, $q^{\prime }$ was changed to 2q−1), and for 1≤q<1.5,

(27) $$S_{q}=\frac{1-{\left(2q-1\right)}^{q}}{1-q}.$$

Let us note that for values of *q* outside the range of variability declared for a given entropy, $S_{q}<1$, i.e., it is always lower than unity, which is less than the Shannon entropy. From Figure 3 it can be seen that the entropy formula $S_{q}$, which could be used in the entire allowable range of the parameter *q*, describing both *q* cases and 2−q dual to them, must contain both elements of (26) and (27), i.e., have the following form:

(28) $$S_{q}=\frac{1}{1-q}\frac{{\left(1-|1-q|\right)}^{q}-1}{q-|1-q|}.$$

The corresponding Tsallis distribution is now

(29) $$f\left(x\right)=\frac{1-\left|1-q^{\prime }\right|}{{\left[1+\left|1-q^{\prime }\right|x\right]}^{\frac{1}{\left|1-q^{\prime }\right|}}}.\;\mathrm{where}\;0.5<q^{\prime }<1.5.$$

A natural question arises as to what should be modified and how in such a case? What we would like to suggest here is the use of appropriately modified definitions of the $\exp_{q}\left(x\right)$ and $\ln_{q}\left(x\right)$ functions, namely to replace $\exp_{q}\left(x\right)$ defined in Equation (2) by

(30) $$\exp_{q}\left(x\right)={\left[1+\kappa x\right]}^{\frac{1}{\kappa }}\;\mathrm{where}\;\kappa =\left(q-1\right)sign\left(x\right)$$

and, accordingly,

(31) $$\ln_{q}\left(y\right)=\frac{y^{\kappa }-1}{\kappa }\;\mathrm{where}\;\kappa =\left(q-1\right)sign\left(y-1\right).$$

**Figure 4:**
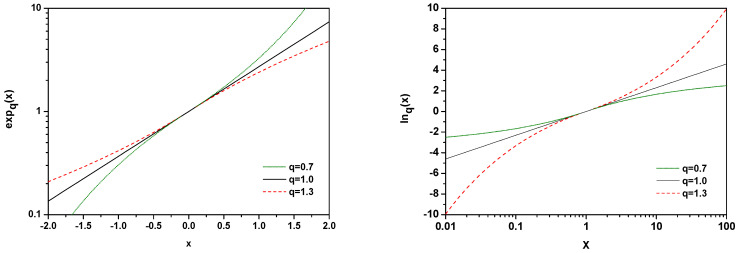
(Color online) Illustration of the behavior of the function $\exp_{q}\left(x\right)$ defined by Equation (30) and the function $\ln_{q}\left(x\right)$ defined by Equation (31) for different values of the parameters *q*.

This form works for all *x* and *q* values, and there are no additional restrictions on the admissible values of the *q* parameter depending on whether x>0 or x<0. Formally, this corresponds to replacing $q\to q^{\prime }=2-q$ when changing the sign of *x*. Figure 4 shows behaviour of the functions $\exp_{q}\left(x\right)$ and $\ln_{q}\left(x\right)$. Note that using this form we now have

(32) $$\exp_{q}\left(-x\right)\cdot \exp_{q}\left(x\right)=1$$

and the ocupation numbers of particles $n_{q}\left(x\right)$ and antiparticles $n_{q}\left(-x\right)$ satisfy relation

(33) $$n_{q}\left(-x\right)+n_{q}\left(x\right)=-\zeta$$

for all values of *q* (ζ=+1 for bosons and −1 for fermions). The naive replecement of the Euler-exponential with another, deformed exponential function (namely given by Equation (2)) can lose the particle-hole symmetry, inherent in the traditional Fermi distribution above and below the Fermi level. Previously, these relationships had a dual form,

(34) $$\exp_{q}\left(-x\right)\cdot \exp_{2-q}\left(x\right)=1\;\mathrm{and}\;n_{q}\left(x\right)+n_{2-q}\left(-x\right)=-\zeta .$$

This means that such an approach avoids not only the problem of duality discussed earlier in Section 2, but also preserves the particle-hole symmetry concerning distribution above and below the Fermi level which is fundamental in field theory and was discussed in [42,43].

In the above considerations, we must remember that the modified functions $\exp_{q}\left(x\right)$ and $\ln_{q}\left(y\right)$ are not differentiable everywhere because the functions sign(x) (in the first case) and sign(1−y) (in the second) have a discontinuity at x=0 or y=1. Therefore, by their derivatives for x=0 (or y=1), we understand their limits for x→0 (or y→1). In this approach, the first derivatives $\exp_{q}\left(x\right)$ and $\ln_{q}\left(y\right)$ are the same for x=0 and y=1 as the first derivatives exp(x) and ln(y), while their *n*-th derivatives already depend on *q* in the following way:

(35) $$\lim_{x\to 0}\frac{d^{n}\exp_{q}\left(x\right)}{dx^{n}}=\prod_{i=1}^{n}\left[i-\left(i-1\right)\right]q]$$

and

(36) $$\lim_{y\to 1}\frac{d^{n}\ln_{q}\left(y\right)}{dy^{n}}=\prod_{i=2}^{n}\left(-i+q\right).$$

## 4. Other Sources of Tsallis Distribution

Note that since Equation (2) describes the data in the entire measured area of phase space, i.e., both those associated with the thermal approach and those associated with hard collisions, the justification of this formula cannot be reduced to the Tsallis entropy only. It is worth noting that for each probability distribution the appropriate form of entropy can be given and for each probability distribution one can also give the constraints which, when used together with the Shannon entropy, lead to this probability distribution [44]. For our considerations, it is important to note that when selecting the constraints in such a way that they best take into account the most important dynamic features of the examined system, one could basically stop at the Shannon entropy [45]. For example, condition 〈x〉=const provides to the usual exponential distribution, $\langle x^{2}\rangle$ gives Gaussian distribution, 〈ln(x)〉=const gamma distribution, whereas $\langle \ln\left(1+x^{2}\right)\rangle$ gives a Cauchy distribution. In general, for some function h(x), the maximum entropy density for f(x) satisfying the constraint ∫dxf(x)h(x)=const has the form $f\left(x\right)=\exp[\lambda_{0}+\lambda h\left(x\right)]$ where parameters $\lambda_{0}$ and λ are fixed by the requirement of normalization for f(x) and by the above constraint. To obtain the Tsallis distribution in this way,

(37) $$f\left(x\right)=\frac{2-q}{x_{0}}{\left[1-\left(1-q\right)\frac{x}{x_{0}}\right]}^{\frac{1}{1-q}}$$

we need to use a constraint like this:

(38) $$\langle \ln\left[1-\left(1-q\right)\frac{x}{x_{0}}\right]\rangle =\frac{q-1}{2-q}.$$

The Tsallis distribution understood as a quasi-power distribution can also be obtained in many ways without referring to any form of entropy [46]. We will now discuss a few of them in more detail.

**Superstatistics**. This approach extends the exponential description, $f\left(E\right)=\frac{1}{T}\exp\left(-\frac{E}{T}\right)$, characterized by some parameter of the scale, *T*, by allowing fluctuations of this parameter [47]. In particular, if they are described by a gamma distribution,

(39) $$g\left(\frac{1}{T}\right)=\frac{1}{\Gamma \left(\frac{1}{q-1}\right)}\frac{T_{0}}{q-1}{\left(\frac{1}{q-1}\frac{T_{0}}{T}\right)}^{\frac{2-q}{q-1}}\exp\left(-\frac{1}{q-1}\frac{T_{0}}{T}\right),$$

the total result is a Tsallis distribution [29,48], the $f_{q}\left(E\right)=\frac{2-q}{T}{\left[1-\left(1-q\right)\frac{E}{T}\right]}^{\frac{1}{1-q}}$, where the parameter *q* characterizing the strength of fluctuations in *T* is given by its variance, $\omega_{T}^{2}=\frac{Var(T)}{{\langle T\rangle }^{2}}=q-1$. Since in thermal models $\omega_{T}^{2}$ is related to the heat capacity $C_{V}$, one possible meaning of the parameter *q* is its relationship to the heat capacity, $q=1+1/C_{V}$ (note that here q>1 always). Other classes of generalized statistics can also be obtained, and with small variance of fluctuations they all behave universally [47].

**Preferential attachment**. This approach describes a situation where the scale parameter depends linearly on the variable under consideration, as is the case when preferential attachment correlations are encountered in the system under consideration, e.g., when $x_{0}\to x_{0}+\left(q-1\right)x$. This changes the equation defining the distribution, resulting in the Tsallis distribution with q>1 [49,50],

(40) $$\frac{df(x)}{dx}=\frac{f(x)}{x_{0}}\to \frac{df(x)}{dx}=\frac{f(x)}{x_{0}+\left(q-1\right)x}\to f\left(x\right)=\frac{2-q}{x_{0}}{\left[1-\left(1-q\right)\frac{x}{x_{0}}\right]}^{\frac{1}{1-q}}.$$

**Tsallis distribution from multiplicative noise**. The Tsallis distribution may also mean that the described process has a stochastic character defined by the additive, γ(t), and multiplicative, ξ(t), noise and described by the Langevin equation,

(41) $$\frac{dp}{dt}+\gamma \left(t\right)p=\xi \left(t\right).$$

The corresponding Fokker-Planck equation has the form

(42) $$\begin{array}{ccc} \frac{\partial f}{\partial t} & = & -\frac{\partial \left(K_{1}f\right)}{\partial p}+\frac{\partial^{2}\left(K_{2}f\right)}{\partial p^{2}}, \end{array}$$

(43) $$\begin{array}{ccc} K_{1} & = & E\left(\xi \right)-E\left(\gamma \right)p\;\mathrm{and}\;K_{2}=Var\left(\xi \right)-2Cov\left(\xi ,\gamma \right)p+Var\left(\gamma \right)p^{2}, \end{array}$$

and for stationary solutions

(44) $$\frac{d\left(K_{2}f\right)}{dp}=K_{1}f.$$

When both noises are uncorrelated (i.e., when Cov(ξ,γ)=0) and when there is no drift caused by additive noise (i.e., E(ξ)=0) the solution to Equation (44) is the Tsallis distribution in $p^{2}$ [51]:

(45) $$f\left(p\right)={\left[1+\left(q-1\right)\frac{p^{2}}{T}\right]}^{\frac{q}{1-q}}\;\mathrm{where}\;T=\frac{2Var(\xi )}{E(\gamma )},\;q=1+\frac{2Var(\gamma )}{E(\gamma )}.$$

The Tsallis distribution with *p* (as in Equation (2)) and not $p^{2}$ is obtained for the more complicated case of T=T(q) when [46]

(46) $$T\left(q\right)=\left(2-q\right)\left[T_{0}+\left(q-1\right)T_{1}\right]\;\mathrm{where}\;T_{0}=-\frac{Cov(\xi ,\gamma )}{E(\gamma )}\;\mathrm{and}\;T_{1}=\frac{E(\xi )}{2E(\gamma )}.$$

Note that *T* now depends non-linearly on *q*, which significantly makes the Tsallis distribution more flexible, allowing for the analysis and comparison of various types of processes (cf. [46]).

At this point, it is worth noting that there is a relationship between the type of noise and the condition imposed in MaxEnt. In the case of Shannon entropy, a condition imposed on the arithmetic mean corresponds to additive noise, while the use of a condition imposed on the geometric mean corresponds to multiplicative noise and leads to a power distribution [52].

**Conditional probability**. The methods for obtaining the Tsallis distribution presented so far are basically limited to cases with q>1. Cases with q<1 can only be observed in constrained systems. Consider for example *N* independent energies, $E_{i=1,\cdots ,N}$, where each of them follows the Boltzman distribution, $g_{i}\left(E_{i}\right)=\frac{1}{\lambda }\exp\left(-\frac{E_{i}}{\lambda }\right)$, and their sum, $E=\sum_{i=1}^{N}E_{i}$, has a gamma distribution, $g_{N}\left(E\right)=\frac{1}{\lambda^{\left(N-1\right)}}{\left(\frac{E}{\lambda }\right)}^{N-1}\exp\left(-\frac{E}{\lambda }\right)$. However, if the available energy is bounded, E=Nα=const, these energies will no longer be independent and will be described by conditional probabilities in the form of Tsallis distributions with q<1:

(47) $$\begin{array}{ccc} f\left(E_{i}|E=N\alpha \right) & = & \frac{g_{1}\left(E_{i}\right)g_{N-1}\left(N\alpha -E_{i}\right)}{g_{N}\left(N\alpha \right)}=\frac{2-q}{\lambda }{\left[1-\left(1-q\right)\frac{E_{i}}{\lambda }\right]}^{\frac{1}{1-q}}, \end{array}$$

(48) $$\begin{array}{ccc} & & \lambda =\frac{\alpha N}{N-1},\;q=\frac{N-3}{N-2}<1. \end{array}$$

One could obtain a Tsallis-like distribution with q>1 only if the scale parameter λ fluctuates in the same way as in the case of superstatistics.

**Statistical physics**. A Tsallis distribution with q<1 also follows from statistical physics. Consider an isolated system with energy U=const and ν degrees of freedom (particles). We choose one of them with energy E≪U, then the rest of the system has energy $E_{r}=U-E$. If this particle is in one well-defined state then the number of states of the entire system is $\Omega (E_{r})$, and the probability that the energy of the selected particle is *E* is P(E)∝Ω(U−E). Expanding lnΩ(U−E) around *U* and keeping only the first two terms one obtains

(49) $$\ln P\left(E\right)\propto \ln\Omega \left(E\right)\propto -\beta E⟹P\left(E\right)\propto e^{-\beta E},$$

that is a Boltzman distribution with

(50) $$\beta =\frac{1}{k_{B}T}\overset{def}{=}\frac{\partial \ln\Omega \left(E_{r}\right)}{\partial E_{r}}.$$

However, it is usually expected that $\Omega \left(E_{r}\right)\propto {\left(\frac{E_{r}}{\nu }\right)}^{\alpha_{1}\nu -\alpha_{2}}$ with $\alpha_{1},\alpha_{2}∼O\left(1\right)$. Choosing $\alpha_{1}=1$ and $\alpha_{2}=2$ (because the number of states in the reservoir has decreased by one), therefore

(51) $$\frac{\partial^{k}\beta }{\partial E_{r}^{k}}\propto {\left(-1\right)}^{k}k!\frac{\nu -2}{E_{r}^{k+1}}={\left(-1\right)}^{k}k!\frac{\beta^{k-1}}{{\left(\nu -2\right)}^{k}}.$$

This allows us to write the probability of selection of energy *E* as:

(52) $$P\left(E\right)\propto \frac{\Omega (U-E)}{\Omega (E)}=C{\left(1-\frac{1}{\nu -2}\beta E\right)}^{\left(\nu -2\right)}=\beta \left(2-q\right){\left[1-\left(1-q\right)\beta E\right]}^{\frac{1}{1-q}},$$

that is, in the form of the Tsallis distribution with $q=1-\frac{1}{\nu -2}\leq 1$, such as in the case of conditional probability above.

## 5. Summary and Conclusions

Entropy has always played an important role in the study of the production mechanisms of particles produced in high-energy hadronic and nuclear collisions, either in their description based on thermodynamics [2] or in descriptions using elements of information theory [4].

In the application of the non-extensive approach, we encounter the problem of a certain duality manifested in the parallel occurrence of the parameter *q* and 2−q, which is best illustrated by the parallel description of particle production processes in nucleon and nuclear collisions discussed in Section 2. The second manifestation of duality appears in an attempt at a non-extensive description of quantum statistical distributions. As suggested by the results of [42,43] they are inconsistent with the conventional description using Tsallis distributions (and prefer the nonextensive Kaniadakis distribution). The point here is the necessity to preserve the particle-hole symmetry requiring that exp(−x)·exp(x)=1, while using the original *q*-exponential Tsallis distribution it leads to $\exp_{q}\left(-x\right)\cdot \exp_{2-q}\left(x\right)=1$. In Section 3 we propose a new formula defining the non-extensive function $\exp_{q}\left(x\right)$ which restores this symmetry and we have a nonextensive version of particle-hole symmetry again which restores this symmetry in the form $\exp_{q}\left(-x\right)\cdot \exp_{q}\left(x\right)=1$.

From a more technical perspective, it is worth noting that both Shannon’s and Tsallis’ entropies have the same generating function, $f\left(x\right)=\sum_{i}p_{i}^{x}$, and that the difference in their forms is just due to the form of adopted differentiation operator. For standard first-order differentiation, df(x)/dx, we obtain the Shannon entropy, whereas adopting the Jackson *q*-derivative, $D_{q}f\left(x\right)=\frac{f(qx)-f(x)}{qx-x}$, yields the Tsallis entropy. In fact, other expressions for entropy can be obtained by using yet other forms of differentiation operators [7].

## Data Availability

Not applicable.
